# Levofloxacin Prophylaxis in Pediatric and Young Adult Allogeneic Hematopoietic Stem Cell Transplantation Recipients Does not Prevent Infective Complications and Infections-related Deaths

**DOI:** 10.1093/ofid/ofae707

**Published:** 2024-12-03

**Authors:** Davide Leardini, Giacomo Gambuti, Edoardo Muratore, Francesco Baccelli, Francesca Gottardi, Francesco Venturelli, Tamara Belotti, Arcangelo Prete, Marco Fabbrini, Patrizia Brigidi, Silvia Turroni, Riccardo Masetti

**Affiliations:** Pediatric Hematology and Oncology, IRCCS Azienda Ospedaliero-Universitaria di Bologna, Bologna, Italy; Pediatric Hematology and Oncology, IRCCS Azienda Ospedaliero-Universitaria di Bologna, Bologna, Italy; Pediatric Hematology and Oncology, IRCCS Azienda Ospedaliero-Universitaria di Bologna, Bologna, Italy; Pediatric Hematology and Oncology, IRCCS Azienda Ospedaliero-Universitaria di Bologna, Bologna, Italy; Pediatric Hematology and Oncology, IRCCS Azienda Ospedaliero-Universitaria di Bologna, Bologna, Italy; Pediatric Hematology and Oncology, IRCCS Azienda Ospedaliero-Universitaria di Bologna, Bologna, Italy; Pediatric Hematology and Oncology, IRCCS Azienda Ospedaliero-Universitaria di Bologna, Bologna, Italy; Pediatric Hematology and Oncology, IRCCS Azienda Ospedaliero-Universitaria di Bologna, Bologna, Italy; Microbiomics Unit, Department of Medical and Surgical Sciences (DIMEC), University of Bologna, Bologna, Italy; Unit of Microbiome Science and Biotechnology, Department of Pharmacy and Biotechnology (FABIT), University of Bologna, Bologna, Italy; Microbiomics Unit, Department of Medical and Surgical Sciences (DIMEC), University of Bologna, Bologna, Italy; Unit of Microbiome Science and Biotechnology, Department of Pharmacy and Biotechnology (FABIT), University of Bologna, Bologna, Italy; Pediatric Hematology and Oncology, IRCCS Azienda Ospedaliero-Universitaria di Bologna, Bologna, Italy; Department of Medical and Surgical Sciences (DIMEC), University of Bologna, Bologna, Italy

**Keywords:** levofloxacin prophylaxis, bacterial infections, febrile neutropenia, gut microbiome, hematopoietic stem cell transplantation

## Abstract

**Background:**

The prophylactic use of quinolones in the setting of allogeneic hematopoietic stem cell transplantation (allo-HCT) is controversial and solid evidence is missing, particularly in children.

**Methods:**

In this single-center retrospective study, we compared outcomes in patients receiving (n = 74) or not receiving (n = 70) levofloxacin (LVX) prophylaxis, assessing overall survival, event-free survival, acute graft-versus-host disease (aGvHD) and bloodstream infection incidence, and infection-related mortality. Gut microbiota composition was analyzed in a subgroup using 16S rRNA sequencing of stool samples collected pre-HCT and at engraftment.

**Results:**

We analyzed 144 allo-HCT in 143 patients performed for any indication. No differences were found in the 2 groups regarding main HCT outcomes, namely, cumulative incidence of aGvHD (37.9% vs 43.5%; *P* = .733), grade III-IV aGvHD (12.2% vs 8.7%; *P* = .469), gut aGVHD (12.2% vs 17.5%; *P* = .451), bloodstream infections (25.6% vs 34.1%; *P* = .236) and death from bacterial infection (9.5% vs 4.3%; *P* = 0.179). In patients experiencing bacterial infections, those receiving prophylaxis showed higher incidence of quinolone-resistant strains (*P* = .001). On a subgroup of 50 patients, we analyzed the gut microbiota composition, showing a lower abundance of *Blautia* (*P* = .015)*, Enterococcus* (*P* = .011), and *Actinomyces* (*P* = .07) at neutrophil engraftment in patients receiving LVX prophylaxis.

**Conclusions:**

LVX prophylaxis in the setting of allo-HCT does not prevent infective complications and increases the prevalence of antibiotic-resistant strains.

Allogeneic hematopoietic stem cell transplantation (allo-HCT) plays a crucial role in the treatment of various malignant and nonmalignant hematological disorders in children [1–4]. Despite significant advances in allo-HCT procedures, infectious complications remain a major cause of morbidity and mortality in this vulnerable population. Bacterial infections pose a significant challenge because of the high risk of severe sepsis and associated mortality [5]. To address this concern, antimicrobial prophylaxis has emerged as a viable strategy to possibly reduce the incidence of infection-related complications and improve overall outcomes in pediatric HCT recipients [6, 7]. In addition, based on preclinical studies from the 1970s, it was hypothesized that total eradication of the intestinal flora prior to transplantation could protect patients from graft versus host disease (GvHD) [8, 9]. Subsequent studies attempted to replicate the findings with heterogeneous results [10–13], and notably, recent evidence suggests that antibiotic exposure may be associated with a higher risk of developing GvHD [14, 15]. Among the various antimicrobial agents available, quinolones have gained considerable attention in recent years. Levofloxacin (LVX), a broad-spectrum fluoroquinolone, exhibits potent activity against a wide range of gram-negative and gram-positive bacteria and offers several advantages, including predictable pharmacokinetics and favorable safety profile. These characteristics make LVX an appealing option for prophylaxis in the HCT setting [16]. The rationale for LVX prophylaxis stems from studies in adult patients with cancer who have demonstrated a significant reduction in bacterial infections and febrile neutropenia, despite conflicting data regarding infection-related mortality [16–18]. The evidence supporting its use in the pediatric population is limited. Importantly, current guideline provides weak recommendations against the routine use of systemic antibacterial prophylaxis in children and young adults undergoing induction chemotherapy for allo-HCT, based on the low level of evidence, and some centers still administer antibacterial prophylaxis [5, 6]. In fact, the unique immunological and physiopathological characteristics of children undergoing allo-HCT warrant a specific evaluation of LVX prophylaxis to determine its efficacy and potential impact on outcomes in this specific cohort. Moreover, there are some concerns about the use of quinolones, including LVX, in pediatric patients because of possible severe adverse drug effects and caution has been advised regarding its use in children unless necessary. In our recent meta-analysis, quinolone prophylaxis was not effective in reducing the rate of bloodstream infections (BSI) in pediatric HCT recipients and associated with a higher rate of bacterial resistance to fluoroquinolones and higher antibiotic exposure [19]. However, as mentioned, the exploration of the topic in children has been somewhat limited. First, the number of patients included is relatively small, and the results in published reports are not fully consistent [19]. Moreover, the included cohorts receiving allo-HCT were often mixed with autologous transplantation, and data on febrile neutropenia and infection-related mortality were scarce [19]. Last, clear data are missing on the effect of quinolone administration on increased antimicrobial resistance and gut microbiota (GM) dysbiosis, which has gained increasing interest in the context of HCT considering its association with transplant outcomes [20–25]. We aim to provide a comprehensive evaluation of LVX prophylaxis in an exclusively pediatric cohort receiving allo-HCT to better characterize its impact on clinical outcomes and GM composition.

## MATERIALS AND METHODS

### Study Population

This single-center retrospective study enrolled patients who underwent allo-HCT for any indication between 2010 and 2022 at the Pediatric Transplant Unit of the IRCCS Azienda Ospedaliero-Universitaria di Bologna. At our institution, patients admitted before May 2016 received antibiotic prophylaxis with intravenous LVX 10 mg/kg/day from the beginning of conditioning to neutrophil engraftment per normal clinical practice. From May 2016, we changed internal antimicrobial stewardship, and no antibacterial prophylaxis was administered, whereas prompt antibiotic treatment was provided in case of the occurrence of febrile neutropenia. Empirical antibiotic therapy strategies also varied over the years. Patients admitted before April 2022 without known colonization by multidrug-resistant strains received ceftazidime as first-line therapy, whereas those admitted from April 2022 onward received piperacillin/tazobactam plus amikacin.

### Study Outcomes and Definitions

The primary outcome of the study was the evaluation of key transplant outcomes in children undergoing allo-HCT, with or without LVX as antibiotic prophylaxis. The analyzed outcomes included overall survival, event-free survival, cumulative incidence of GvHD, BSI, and mortality from infections or any cause. Secondary outcomes included the analysis of pathogens and their antibiotic resistance profiles in both groups, as well as changes in GM composition. Neutropenia was defined as an absolute neutrophil count below 0.5 × 10^9^/L and fever was defined as a body temperature ≥ 38 °C from 2 separate determinations. BSI episodes were defined as positive blood cultures from peripheral blood with or without a positive culture for the same bacteria from the central venous catheter including bacteria commonly considered as commensals. The differential time to positivity was considered to distinguish catheter-related BSI. In the case of a positive blood culture, an antibiogram was obtained to investigate antibiotic resistance. A multidrug-resistant (MDR) strain was defined as resistant to at least 1 agent in 3 or more antimicrobial classes. For a further characterization of BSI episodes and to investigate the origin of bacterial infections, we followed the National Healthcare Safety Network (NHSN) classification and criteria. This classification divides BSI into mucosal barrier injury laboratory-confirmed bloodstream infections (MBI-LCBI), BSI other, and MBI-LCBI + BSI other, as described in Dandoy et al. [26]. BSI other was defined as BSI not related to indwelling catheters or infections at other sites, as previously reported [26]. The NHSN adopted this classification to identify a specific group of BSIs reported as central line–associated, which are related to mucosal barrier injury rather than the presence of a central venous catheter. The retrospective study was approved by the Ethics Committee of Sant’Orsola-Malpighi University Hospital (ref. number 868/2020/Oss/AOUBo). The microbiome study was approved by the Ethics Committee of Sant’Orsola-Malpighi University Hospital (ref. number 19/2013/U/Tess) and each patient signed the informed consent to participate in the study.

### Microbiome Analysis

Analysis was performed on a subgroup of patients included in these retrospective studies and some of the included patients were previously enrolled also in Masetti et al. [25]. Patients with available stool samples collected before HCT and at the time of engraftment were considered eligible. New analyses were conducted on these previously reported patients. The patients included in the analyses were transplanted between 2012 and 2020. Full methods of microbiome collection and analysis is reported in Masetti et al. In brief, stool samples were collected in sterile tubes and stored at a temperature of –80 °C, before microbiological analysis was performed. Total microbial DNA was extracted and the 16S rRNA hypervariable region V3-V4 was amplified and sequenced on an Illumina MiSeq platform with a 2 × 250-bp paired-end protocol. Raw reads were processed using a pipeline combining PANDASeq [27] and QIIME2 [28]. After length and quality filtering, the reads were binned into amplicon sequence variants with DADA2 [29]. Taxonomic assignment was carried out implementing a hybrid sklearn q2-feature-classifier [30] in QIIME2 against the SILVA 138 SSURef NR99 [31] database processed with RESCRIPt [32]. Alpha diversity and relative abundance amplicon sequence variants tables were computed with QIIME2.

### Statistical Analysis

Qualitative clinical variables were reported as number and percentage of the total and compared using Fisher exact test. Continuous variables were reported as median and interquartile range and compared using the Mann–Whitney *U* test. The cumulative incidence of BSI and HCT complications after HCT was calculated using the method of Kalbfleisch and Prentice and compared with the Gray test. Both outcomes were calculated at 100 days after HCT. Crude number of infections was calculated considering the infections during the neutropenic period. We used the Fine-Gray model to account for competing risks and performed propensity score matching to adjust for covariates and time-related factors. We then fitted the Fine-Gray model on the matched data and extracted the significance and confidence intervals from the Fine-Gray model fit (using the *coxph* function from the survival R package v3.7-0). All *P* values were calculated with the 2-sided method, and values lower than .05 were considered statistically significant. Analysis was performed using NCSS 12 Statistical Software (2018; NCSS, LLC, Kaysville, Utah, USA). Microbiota analyses were conducted in R 4.3.1. All plots were generated with the microbAIDeR R-package [33]. Differences in alpha diversity were tested with Kruskal-Wallis tests followed by post hoc pairwise Wilcoxon tests. Beta diversity distances were computed from the genera-level relative abundance out table with Bray-Curtis dissimilarity index. Differences in beta diversity were assessed using permutational analysis of variance testing with the pairwise.adonis function from pairwiseAdonis, implemented in the microbAIDeR pipeline [34]. All *P* values have been corrected for multiple testing with the Benjamini-Hochberg false discovery rate (FDR). °*P* value < .1 was considered as a trend; *P value ≤ .05; ***P value ≤ 0.001.

## RESULTS

### Patient Characteristics

The study enrolled 143 patients for a total of 144 allo-HCT. The median age of the cohort was 9 years (range, 4 months–23 years). The most common indication for HCT were acute lymphoblastic leukemia (n = 53; 36.8%) and acute myeloid leukemia (n = 44; 30.5%), whereas 33 patients had nonmalignant disease, mainly beta thalassemia (n = 11; 7.6%) and aplastic anemia (n = 9; 6.2%). Among patients with leukemia, 55 (38.2%) were in first remission while 42 (29.2%) in second or more remission. Patients received HCT from matched unrelated donors, matched sibling donors, and haploidentical donors in 93 (64.6%), 29 (20.1%), and 22 (15.3%), respectively. The source of HSCs was bone marrow in 118 patients (81.9%), peripheral blood stem cells in 18 (12.5%), and cord blood in 8 (5.5%). Most patients received myeloablative conditioning (n = 130, 90.3%) and among them 15 (11.5%) received total body irradiation. One hundred and thirty-one patients (91.0%) received granulocyte colony stimulating factor. Seventy-four patients (51.4%) received LVX prophylaxis, whereas 70 (48.6%) did not. The clinical and transplant features of the 2 groups are summarized in Table 1.

**Table 1.** Clinical Characteristic of the Patients Included in the Study

Characteristic	Exposed to LVX (%) (N = 74)	Not Exposed to (%) LVX (N = 70)	*P* Value
**Age at HCT, y (range)**	9 (0.7–23.0)	9 (0.3–21.0)	.372
**Male sex, no. (%)**	34 (45.9)	36 (51.4)	.510
**Malignant, no. (%)**	56 (75.7)	55 (78.6)	.679
**Indication, no. (%)**	…	…	.351
AML	24 (32.4)	20 (28.6)	
I CR	19 (25.7)	15 (21.4)	
≥II CR	5 (6.7)	5 (7.2)	
ALL	28 (37.8)	25 (35.7)	
I CR	13 (17.6)	8 (11.4)	
≥II CR	15 (20.2)	17 (24.3)	
Other malignant	4 (5.4)	10 (14.3)	
Nonmalignant	18 (24.4)	15 (21.4)	
**Donor, no. (%)**	…	…	.**001**
MUD	45 (60.8)	48 (68.6)	
9/10	1 (2.2)	3 (6.3)	
10/10	44 (97.8)	45 (93.7)	
Haplo	6 (8.1)	16 (22.9)	
MRD	23 (31.1)	6 (8.6)	
**Sex mismatch, %**	38%	38%	1.000
**Graft type, no. (%)**	…	…	.038
BM	61 (82.4)	57 (81.4)	
PBSC	6 (8.1)	12 (17.2)	
CB	7 (9.5)	1 (1.4)	
**Intensity of conditioning, no. (%)**	…	…	.913
MAC	67 (90.5)	63 (90.0)	
TBI	9 (13.4)	5 (7.9)	
RIC	7 (9.5)	7 (10.0)	
**Remission at HCT, no. (%)**	…	…	.906
Yes	41 (55.4)	41 (58.6)	
No	15 (20.3)	14 (20.0)	
Not evaluable	18 (24.3)	15 (21.4)	
**ATG, no. (%)**	49 (66.2)	50 (71.4)	.500
**GvHD prophylaxis**	…	…	.002
CNI only	19 (25.7)	4 (5.7)	
CNI + ATG + MTX	44 (59.4)	44 (62.9)	
Other regimes	11 (14.9)	21 (30.0)	
**Granulocyte colony stimulating factor, no. (%)**	69 (93.2)	62 (88.6)	.328
**Primary graft failure, no. (%)**	7 (9.4)	4 (5.7)	.534
**Time to neutrophils engraftment, days, median (range)**	14 (8–27)	16 (11–32)	.**018**
**Patients followed until day +100 from HCT, no. (%)**	56 (75.7)	57 (81.4)	.401

Values in bold mean that the *P*-value is inferior to .05.

Abbreviations: ALL, acute lymphoblastic leukemia; AML, acute myeloid leukemia; ATG, anti-thymocyte globulin; BM, bone marrow; CB, cord blood; CNI, calcineurin inhibitor; GvHD, graft versus host disease; HCT, hematopoietic stem cell transplantation; MAC, myeloablative conditioning; MRD, matched related donor; MUD, matched unrelated donor; MTX, methotrexate; PBSC, peripheral blood stem cells; RIC, reduced intensity conditioning.

### HCT Outcomes in Patients Receiving or not LVX Prophylaxis

**Figure 1.**
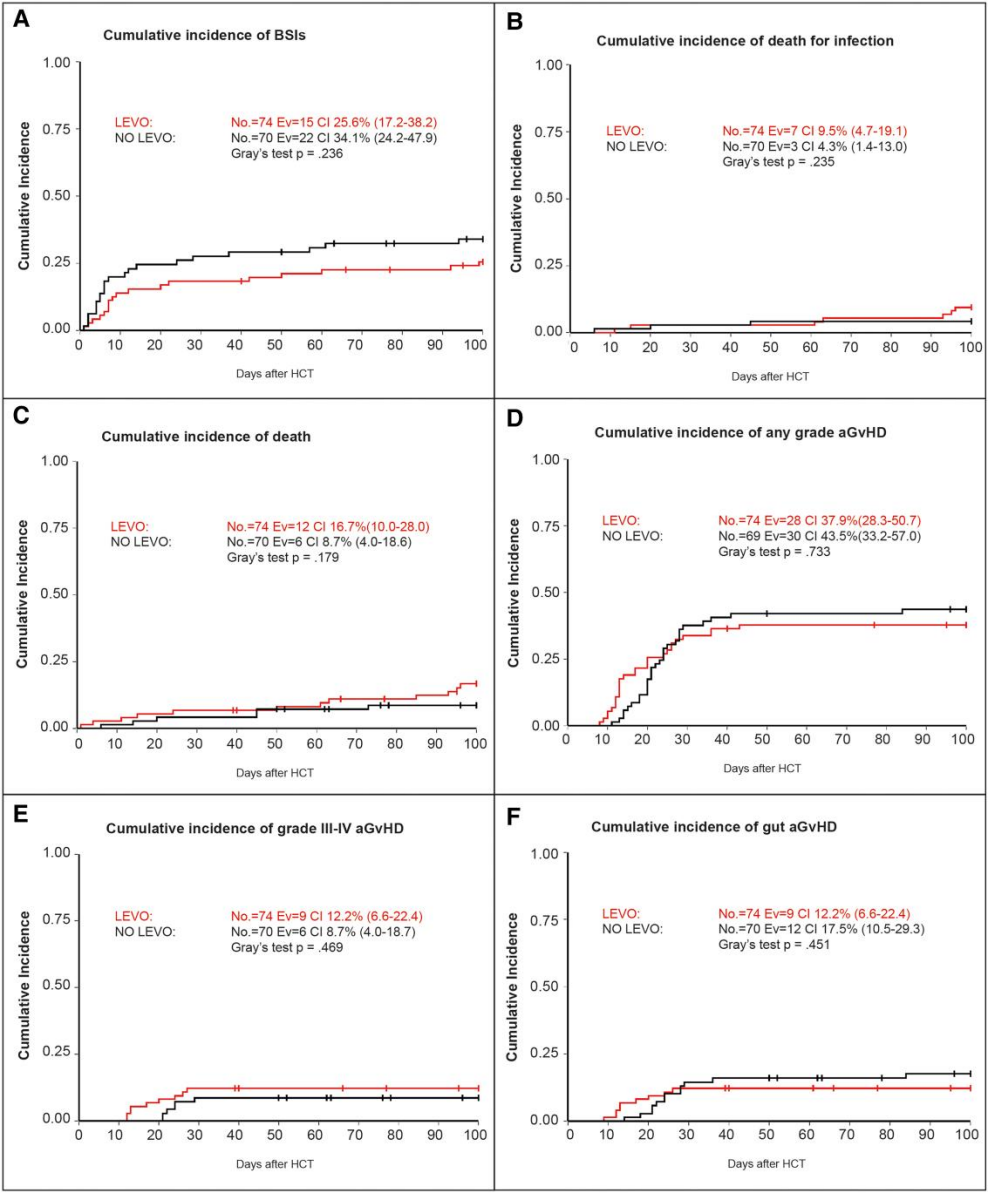
Main HCT outcome in patients receiving and not receiving LVX prophylaxis. Cumulative incidence of (*A*) bloodstream infections, (*B*) death for bacterial infections within 100 days after HCT, (*C*) death within 100 days after HCT, (*D*) any grade acute GvHD, (*E*) grade III-IV acute GvHD, and (*F*) gut GvHD. CI, cumulative incidence; Ev, events; LVX, levofloxacin prophylaxis.

The comparison between the main HCT outcomes at 100 days after HCT of the patients receiving and not receiving LVX prophylaxis is reported in Figure 1. Overall, we found no differences in patients receiving and not receiving LVX in terms of 100 days’ cumulative incidence of BSI (25.6%; 95% confidence interval [CI], 17.2–38.2) vs 34.1% [95% CI, 24.2–47.9]; *P* = 0.236) as well as in the 30-day cumulative incidence of BSI (18.3% [95% CI, 11.2–29.9) vs 27.7% [95% CI, 18.7–41.0]; *P* = .364). Calculating the crude BSI rate in the 2 groups, we found no differences as well (20.3% vs 31.4%, *P* = .126). The 2 groups did not present any differences also in the 100-day cumulative incidence of any grade acute GvHD (aGvHD; 37.9% [95% CI, 28.3–50.7%] vs 43.5% [95% CI, 33.2–57.0]; *P* = .733), grade II-IV aGvHD (31.3% [95% CI, 22.2–43.7] vs 30.6% [95% CI, 21.4–43.7]; *P* = .469), grade III-IV aGvHD (12.2% [95% CI, 6.6–22.4] vs 8.7% [95% CI, 4.0–18.7%]; *P* = .469), and gut GvHD (12.2% [95% CI, 6.6–22.4] vs 17.5% [95% CI, 10.5–29.3%]; *P* = .451). No differences were found in the 2 groups regarding the 100-day cumulative incidence of death for any cause (16.7% [95% CI, 10.0–28.0] vs 8.7% [95% CI, 4.0–18.6]; *P* = .179) and death from bacterial infections (9.5% [95% CI, 4.7–19.1] vs 4.3% [95% CI, 1.4–13.0]; *P* = .179). Overall survival and event-free survival at 2 years for patients receiving and not receiving LVX prophylaxis was 66.0% (95% CI, 54.6–77.4) vs 56.9% (95% CI, 38.4–75.4) (*P* = .757) and 62.8% (95% CI, 51.2–74.5%) vs 51.7% (95% CI, 32.4–71.1) (*P* = .747), respectively. Detailed information regarding the causes of death is reported in Supplementary Table 1, including the type of bacteria with the associated resistance profile. The absence of an effect of LVX prophylaxis was also demonstrated in a model adjusted by age, intensity of conditioning regimen, graft source, and type of donor. To reduce bias related to the timespan during which patients received HCT, we applied covariate adjustment using transplant year and patient matching based on propensity score methods. We did not find any significant association between LVX prophylaxis and the main transplant outcomes (Supplementary Table 2).

### Prophylaxis With LVX and BSI

We then assessed the characteristics of BSI developed in the neutropenic phase in the group receiving and not receiving LVX (Table 2). BSI episodes during neutropenia in the group receiving and not receiving LVX prophylaxis affected 15 and 22 patients, respectively, with no significant difference in organism classification: Gram-negative (46.7% vs 54.5%, *P* = .638), Gram-positive (66.7% vs 54.5%, *P* = .461) or both Gram-negative and Gram-positive (13.3% vs 9.1%, *P* = 1.000). The proportion of BSI caused by *Viridans streptococci*, for which LVX is particularly active, did not significantly change between the 2 groups (26.7% vs. 13.6%, *P* = .320). Notably, resistance to LVX in the exposed patients was 100%, whereas it decreased to 0% in the nonexposed cohort (*P* = .010). The BSI caused by MDR microorganisms affected 86.7% and 54.5% of patients, respectively (*P* = .073), with isolates resistant to quinolones in 80.0% vs 22.7% of patients (*P* = .001) and bacteria resistant to beta lactams in 53.3% vs 50.0% of patients (*P* = .842). There was also a significant difference in BSI caused by Gram-positive bacteria resistant to quinolones (60.0% vs 9.1%, *P* = .001). The episodes of BSI were classified according to NHSN criteria, with no difference in the type of bacterial infection between the 2 groups: MBI-LCBI (80.0% vs 50.0%, *P* = .091), BSI other (13.3% vs 27.3%, *P* = .431), and MBI-LCBI + BSI other (6.7% vs 22.7%, *P* = .368). The specific characteristics of each patient's BSI events are illustrated in Supplementary Table 3 and Supplementary Table 4. We also analyzed the median length of antibiotic treatment during the neutropenic phase in patients receiving and not receiving LVX prophylaxis and we found no statistically significant differences (15.9 vs 18.8 days, *P* = .057). We also analyzed the days of fever in the first 30 days after HCT, patients receiving and not receiving LVX prophylaxis presented a median duration of fever of 7.5 (range, 0–26) and 9.0 (range, 0–29) (*P* = .290).

**Table 2.** Detailed Characteristics of BSI in the 2 Groups

BSI Details	Exposed to LVX (No. = 15)	Not Exposed to LVX (No. = 22)	*P* Value
**BSI Before Engraftment**			
Gram–	7 (46.7)	12 (54.5)	.638
Gram+	10 (66.7)	12 (54.5)	.461
Gram–, Gram+	2 (13.3)	2 (9.1)	1
Multidrug resistant	13 (86.7)	12 (54.5)	.073
Resistant to Qs	12 (80.0)	5 (22.7)	**.001**
Resistant to BLs	8 (53.3)	11 (50.0)	.842
**Isolated bacteria**			
Gram-negative resistant to Qs	4 (26.7)	4 (18.2)	.538
Gram-negative resistant to BLs	5 (33.3)	8 (36.4)	.850
Gram-positive resistant to Qs	9 (60.0)	2 (9.1)	**.001**
Gram-positive resistant to BLs	4 (26.7)	4 (18.2)	.538
Gram-negative, positive resistant to Qs	2 (13.3)	1 (4.5)	.554
Gram-negative, positive resistant to BLs	2 (13.3)	1 (4.5)	.554
*Streptococci viridans*	4 (26.7)	3 (13.6)	.320
*Streptococci viridans*, resistant to Qs	4 (26.7)	0 (0)	**.010**
**NHSN classification**			
MBI-LCBI	12 (80.0)	11 (50.0)	.091
BSI other	2 (13.3)	6 (27.3)	.431
MBI-LCBI + BSI other	1 (6.7)	5 (22.7)	.368

Abbreviations: BSI, blood stream infection; BL, beta-lactam; CR, complete remission; MBI-LCBI, mucosal barrier injury laboratory confirmed bloodstream infection; Q, quinolones.

### Microbiota Analysis

**Figure 2.**
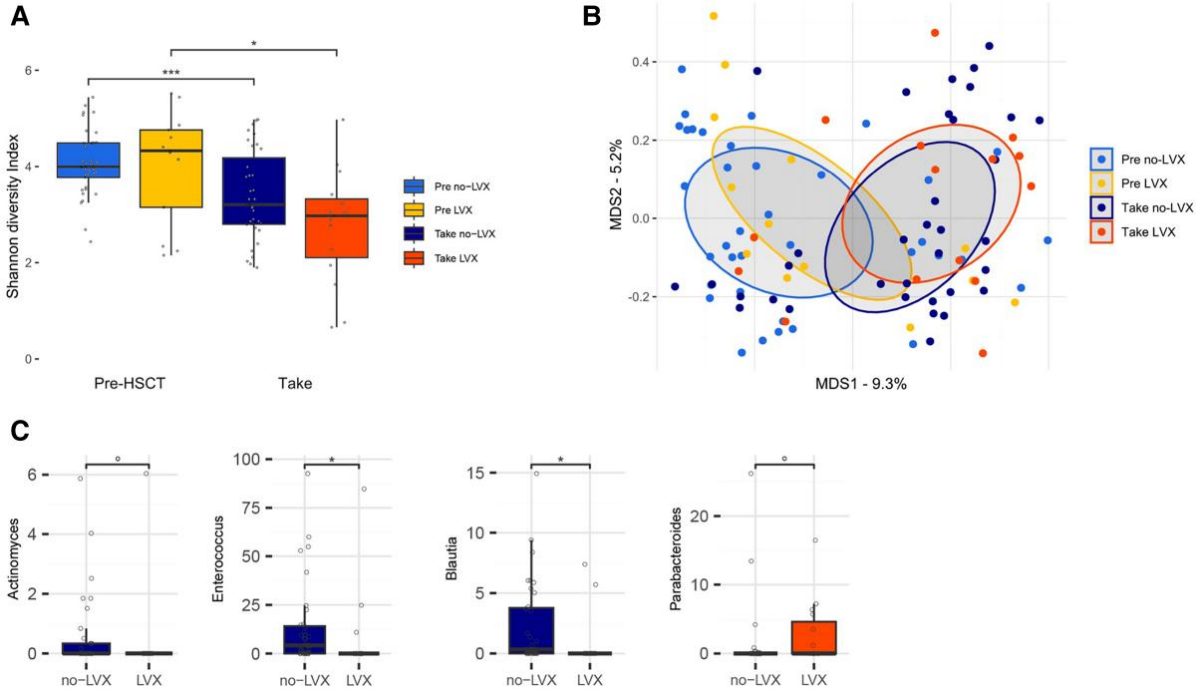
Gut microbiota in pediatric HCT evaluating the impact of LVX prophylaxis. (*A*) Boxplots showing alpha diversity values according to Shannon's Index according to LVX prophylaxis and transplant time course. TAKE: Neutrophil engraftment. (*B*) Bray-Curtis dissimilarity principal coordinate analysis (PCoA) plot. Ellipses represent the 95% confidence area based on the standard error of the weighted average of samples coordinate. MDS: MultiDimensional scaling. (*C*) Genera-level differences at neutrophil engraftment between the patients receiving and not receiving LVX prophylaxis. Significance bars represent post hoc pairwise Wilcoxon tests with false discovery rate (FDR) correction for multiple testing (°*P* value < 0.1 was considered as a trend; *P value ≤ 0.05; ***P value ≤ 0.001).

GM diversity according to Shannon's Index revealed no significant differences before HCT in patients receiving or not receiving LVX prophylaxis (Figure 2*A*, *P* = .520). Both patient groups receiving and not receiving LVX prophylaxis showed a significant reduction in alpha diversity at neutrophil engraftment (*P* = .013 and *P* < .001, respectively). The percentage reduction in alpha diversity between pre-HCT and neutrophil engraftment for the 2 groups is comparable: the prophylaxis group showed a 26.0 ± 9.9% (standard error [SE]) reduction in Shannon's index, whereas the reduction settles to 18.0 ± 8.1% for the group not receiving LVX prophylaxis. Beta diversity principal coordinate analysis plot (Figure 2*B*) computed from Bray-Curtis dissimilarity matrices showed that the strongest differences in microbiota composition are linked to the transplant timepoint (permutational analysis of variance testing, FDR < 0.03). Before transplant, no significant differences between the two groups in terms of taxa relative abundances were found. Nonetheless, we detected significant differences at neutrophil engraftment (Figure 2*C*), with patients receiving LVX prophylaxis showing a trend of higher relative abundances of *Parabacteroides* (*P* = .09) and lower abundances of *Blautia* (*P* = .015)*, Enterococcus* (*P* = .011), and *Actinomyces* (*P* = .07).

## DISCUSSION

In this single-center study, we conducted a comparative analysis of pediatric and young adult patients undergoing HCT who either received or did not receive LVX prophylaxis. We also conducted a comprehensive clinical and microbiological assessment of these patients and observed no significant impact on the incidence of BSI and death related to bacterial infections. These data contribute to the relatively limited evidence on the role of antibacterial prophylaxis in pediatric HCT by combining, for the first time, clinical and microbiological outcomes in a large pediatric cohort, suggesting against its routine use in this setting. Indeed, the use of prophylactic antibiotics has been linked to potential clinical benefits in reducing post-HCT bacterial infections while modulating negatively the occurrence of GvHD. Moreover, it also poses a heightened risk of bacterial resistance development. Striking a balance between these risks and benefits is challenging in the pediatric setting, where available data are limited and heterogeneous, as noted in our recent meta-analysis [19]. Our study did not reveal any significant influence of LVX prophylaxis on the primary clinical outcomes of HCT. These findings align with previous pediatric studies on HCT recipients, which similarly reported no differences in terms of survival and mortality between patients who received quinolone prophylaxis and those who did not [35, 36]. Notably, although Gardner et al. documented a higher rate of aGvHD in their prophylaxis group, we did not observe any significant differences in the occurrence of any grade, severe, or gut GvHD in our patient cohort [13]. We did, however, identify a higher percentage of BSI cases caused by resistant bacteria in patients who received prophylaxis. Specifically, resistance to quinolones was significantly elevated, and there was a trend toward a higher proportion of MDR etiologic agents in the LVX group, although the latter did not reach statistical significance. The results are also striking considering the shift in the resistance pattern in *Streptococc*i *viridans.* These findings align with a pediatric study that reported a significant increase in quinolone resistance among prophylaxis-receiving patients, whereas a larger study by Alexander et al. found no significant difference in LVX resistance [36, 37]. The emergence of new antibiotic resistance in bacterial microflora is a critical concern in cancer patients, and this effect has been noted in studies on quinolone prophylaxis, especially with fourth-generation cephalosporins, which are first-line antibiotics for managing febrile neutropenia [5, 16, 38, 39]. Regarding GM composition, both prophylaxis and nonprophylaxis groups exhibited homogeneity in alpha diversity before HCT and before the start of LVX prophylaxis. Both groups experienced a significant reduction in diversity at neutrophil engraftment, indicating that the transplant procedure itself, compounded by the conditioning regimen and standard supportive care, plays a substantial role. The LVX prophylaxis group exhibited a trend towards greater reduction in diversity during HCT, which may be attributed to the previously reported adverse impact of LVX prophylaxis on microbiome resistance and plasticity in pediatric HCT patients [24]. Consistent with the alpha diversity results, a pronounced shifts in beta diversity occurred between the pre-HCT and engraftment timepoints, underscoring once again the significant influence of the transplant procedure itself on gut microbiota. Although there were no significant differences in GM composition before transplant between the prophylaxis and nonprophylaxis groups, taxonomic studies revealed species-level differences after HCT. At neutrophil engraftment, several compositional differences emerged between the prophylaxis groups, with the LVX group showing a decreased levels of *Blautia* and *Enterococcus*. Despite being an opportunistic pathogen and a known pathobiont associated with worse clinical outcomes in the HCT setting, lower levels of *Enterococcus* in the LVX group were not associated with a prevention of infectious complications or a lower incidence of GvHD [40, 41]. On the other side, decreased levels of *Blautia* have been associated with higher incidence and severity of aGvHD [42–45]. However, our data did not reveal a clear correlation between antibiotic prophylaxis and aGvHD, possibly because of the limited impact of LVX on GM. Even without clinical consequences, it is possible to speculate that that LVX may exert a selective effect on susceptible bacterial population such as *Blautia* and *Enterococcus.* In our cohort, we also observed a trend toward a decrease in *Actinomyces* and an increase in *Parabacteroides*, with the latter being a member of the *Bacteroidetes* phylum. Both taxa have previously been associated with a lower incidence of GvHD in pediatric cohorts [46, 47] and depletion of Bacteroidetes has been recently associated with steroid-resistant gut GvHD in adults [48]. Indeed, the specific associations between antibiotics, GM alterations, and clinical outcomes should be explored further in larger cohorts to better define the role of each antibiotic and the extent of GM alterations. In particular, GM studies in HCT should focus on defining specific microbial patterns that predict an increased risk of transplant complications and response rate to treatments to stratify patients at risk and to focus on the impact of single interventions to these patterns [21]. Such investigations could aid clinicians in making informed decisions about antibiotic therapies in the HCT setting [23]. Moreover, microbiome-directed interventions, such as fecal microbiota transplantation or prebiotics, could prevent detrimental microbial alterations during HCT and improve clinical outcomes [45, 49, 50]. Our study presents some limitations that include the retrospective nature of the study and the relatively long timeframe in which patients were treated, which resulted in some heterogeneity of some of the patients' characteristics, namely, the type of donor and the GvHD prophylaxis.

## CONCLUSIONS

In summary, our study demonstrates that the prophylactic use of LVX in pediatric and young adult patients undergoing HCT does not effectively prevent infectious complications and bacterial infection–related deaths. Notably, LVX prophylaxis is associated with a substantial increase in the prevalence of antibiotic-resistant strains. Additionally, LVX prophylaxis appears to exert a discernible impact on the composition of the GM at the genus level, but these alterations do not translate into clinically significant correlations. Taken together, our results provide further support for the recommendation of avoiding the routine use of quinolone prophylaxis agents in the context of pediatric and young adult HCT.

## Supplementary Material

ofae707_Supplementary_Data

## Supplementary Data

Supplementary materials are available at *Open Forum Infectious Diseases* online. Consisting of data provided by the authors to benefit the reader, the posted materials are not copyedited and are the sole responsibility of the authors, so questions or comments should be addressed to the corresponding author.

## Notes

***Acknowledgments.*** The work reported in this publication was funded by the Italian Ministry of Health, RC-2024-2790372, and was supported by the Fondazione Cassa di Risparmio di Bologna (CARISBO) [grant number 19431–2021.0082 to R.M.].

***Author contributions.*** D.L., E.M., F.B., and R.M. conceptualized the study; D.L., E.M., F.B., F.G., and G.G. collected the data; M.F. and S.T. performed the microbiological analysis; D.L. and G.G. performed the statistical analysis; M.F. and D.L. prepared the figures; D.L., E.M., M.F., F.B., F.G., and G.G. wrote the paper; R.M., S.T., and P.B. critically reviewed the paper.

***Data availability statement.*** The datasets used and analyzed during the current study available from the corresponding author on reasonable request.
