# Supplementary material for: Levofloxacin Prophylaxis in Pediatric and Young Adult Allogeneic Hematopoietic Stem Cell Transplantation Recipients Does not Prevent Infective Complications and Infections-related Deaths
Source: Open Forum Infect Dis. 2024 Dec 3;12(2):ofae707. doi: 10.1093/ofid/ofae707 (PMC11811901; doi:10.1093/ofid/ofae707)
Supplement: ofae707_Supplementary_Data [file ofae707_supplementary_data.docx]

**Levofloxacin prophylaxis in pediatric and young adults allogeneic hematopoietic stem cell transplantation recipients does not prevent infective complications and infections-related deaths and modifies the gut microbiome**

Davide Leardini^1^, Giacomo Gambuti^1^, Edoardo Muratore^1,*^, Francesco Baccelli^1^, Francesca Gottardi^1^, Francesco Venturelli^1^, Tamara Belotti^1^, Arcangelo Prete^1^, Marco Fabbrini^2,3^, Patrizia Brigidi^2^, Silvia Turroni^3^, Riccardo Masetti^1,4^

^1^ Pediatric Hematology and Oncology, IRCCS Azienda Ospedaliero-Universitaria di Bologna, Bologna, Italy;

^2^ Microbiomics Unit, Department of Medical and Surgical Sciences (DIMEC), University of Bologna, Bologna, Italy;

^3^ Unit of Microbiome Science and Biotechnology, Department of Pharmacy and Biotechnology (FABIT), University of Bologna, Bologna, Italy;

^4^ Department of Medical and Surgical Sciences (DIMEC), University of Bologna, Bologna, Italy.

* corresponding author

**Supplementary Information**

**Supplementary Table 1**: Causes of death in the two groups. * Four out of five of the BSI isolates were resistant to levofloxacin, while one was non-resistant; data for two isolates is unavailable § none of the BSI isolates were resistant to levofloxacin

|  | **Exposed to levofloxacin (N=74)** | **Not exposed to levofloxacin (N=70)** | **P value** |
| --- | --- | --- | --- |
| **Causes of death – % (n.)** Disease progression Infection related mortality  Bacterial infection  Viral infection  Fungal infection Other | 7.7% (1) 69.2% (9) 77.8% (7)*  22.2% (2)  0% (0)  23.1% (3) | 16.7% (1) 66.6% (4) 75.0% (3)§  25.0% (1)  0% (0)  16.7% (1) | 0.819 |

**Supplementary Table 2:** Association between LVX prophylaxis and main transplant outcomes after covariate adjustment and propensity score matching

|  |  | **After covariate adjustment** | | | |  | **After propensity score matching** | | |
| --- | --- | --- | --- | --- | --- | --- | --- | --- | --- |
|  |  | p value | 95% CI  Lower limit | 95% CI  Upper limit |  | | p value | 95% CI  Lower limit | 95% CI  Upper limit |
| BSI at 30 days |  | 0.932 | 0.29- | 3.91 |  | | 0.413 | 0.13 | 2.31 |
| BSI at 100 days |  | 0.977 | 0.26- | 3.67 |  | | 0.39 | 0.15 | 2.12 |
| aGvHD II-IV |  | 0.963 | 0.28- | 3.77 |  | | 0.92 | 0.25 | 4.57 |
| aGvHD III-IV |  | 0.218 | 0.03- | 2.27 |  | | 0.286 | 0.03 | 2.83 |
| Gut GvHD |  | 0.401 | 0.04- | 3.54 |  | | 0.267 | 0.03 | 2.72 |
| Overall survival |  | 0.119 | 0.12- | 1.27 |  | | 0.331 | 0.15 | 1.9 |

**Supplementary Table 3**: Detailed characteristics of BSI in patients receiving LVX prophylaxis.

| **Patient** | **Days post-HCT of blood culture collection** | **Microrganism** | **Resistance to quinolones** | **Resistance to**  **b-lactams** | **NHSN organisms list** | **Classification** |
| --- | --- | --- | --- | --- | --- | --- |
| **L_01** | 2 | *S. Epidermidis* | Yes | Yes | ALL/CC/UTI | MBI-LCB2 |
| **L_02** | 7 | *E. Coli* | Yes | Yes | ALL/MBI/UTI | MBI-LCB1 |
| **L_03** | 0 | *S. Pneumoniae* | Yes | No | ALL/UTI | BSI other |
| **L_04** | 9 | *S. Mitis - S.Pneumoniae* | Yes (S. mitis) | Yes (S. mitis) | ALL/CC/MBI/UTI - ALL/UTI | MBI-LCB1 + BSI other |
| **L_05** | 0 | *E. Coli* | No | Yes | ALL/MBI/UTI | MBI-LCB1 |
| **L_06** | 12 | *Stafilococco* | Yes | No | ALL/UTI | BSI other |
| **L_07** | 7 | *Gemella haemolysan* | Yes | No | ALL/MBI/UTI | MBI-LCB1 |
| **L_08** | 1 | *K. Pneumoniae - E. Fecalis* | Yes (K. pneumoniae) | Yes (K. pneumoniae) | ALL/MBI/UTI - ALL/MBI/UTI | MBI-LCB1 |
| **L_09** | 5 | *S. Epidermidis - E. Coli - S. mitis* | Yes (S. mitis, S. epidermidis, E. coli) | Yes (S. epidermidis, E. coli) | ALL/CC/UTI - ALL/MBI/UTI - ALL/CC/MBI/UTI | MBI-LCB1 |
| **L_10** | 0 | *E. Coli* | Yes | Yes | ALL/MBI/UTI | MBI-LCB1 |
| **L_11** | 20 | *S. Haemolyticus* | Yes | Yes | ALL/CC/UTI | MBI-LCB2 |
| **L_12** | 3 | *F. Nucleatum - L. Buccalis* | No | No | ALL/MBI/UTI - ALL/MBI/UTI | MBI-LCB1 |
| **L_13** | 7 | *S. Mitis* | Yes | No | ALL/CC/MBI/UTI | MBI-LCB1 |
| **L_14** | 6 | *S. Mitis* | Yes | No | ALL/CC/MBI/UTI | MBI-LCB1 |
| **L_15** | 8 | *E. Coli* | No | No | ALL/MBI/UTI | MBI-LCB1 |

**Supplementary Table 4**: Detailed characteristics of BSI in patients non receiving LVX prophylaxis.

| **Patient** | **Days post-HCT of blood culture collection** | **Microrganism** | **Resistance to quinolones** | **Resistance to**  **b-lactams** | **NHSN organisms list** | **Classification** |
| --- | --- | --- | --- | --- | --- | --- |
| **NL_01** | 0 | *Streptococco disgalactiae* | No | No | ALL/UTI | BSI other |
| **NL_02** | 4 3 10 | *MSSA e micrococcus luteus; s. hominis, e avium, s. parasanguinis* | No | No | ALL/UTI - ALL/CC/UTI - ALL/CC/UTI - ALL/MBI/UTI - ALL/CC/MBI/UTI | MBI-LCBI1 + BSI other |
| **NL_03** | 2 | *S. Mitis* | No | No | ALL/CC/MBI/UTI | MBI-LCB1 |
| **NL_04** | 2 | *Enterobacter Aerogenes* | No | Yes | ALL/MBI/UTI | MBI-LCB1 |
| **NL_05** | 4 | *S. Aureus* | No | No | ALL/UTI | BSI other |
| **NL_06** | 6 | *E. Coli* | Yes | Yes | ALL/MBI/UTI | MBI-LCB1 |
| **NL_07** | 2 | *Campilobacter coli* | Yes | No | ALL/UTI | BSI other |
| **NL_08** | 0 | *E. coli* | No | Yes | ALL/MBI/UTI | MBI-LCB1 |
| **NL_09** | 24 | *S mitis (viridans strept)* | No | No | ALL/CC/MBI/UTI | BSI other |
| **NL_10** | 12 | *E. cloacae CPE VIM* | No | Yes | ALL/MBI/UTI | MBI-LCB1 |
| **NL_11** | 11 | *Capnocitophaga sputigena* | No | No | ALL/MBI/UTI | MBI-LCB1 |
| **NL_12** | 1 | *E. coli, K. Pneumoniae* | Yes (E. coli) | Yes (E. coli, K. pneumoniae) | ALL/MBI/UTI - ALL/MBI/UTI | MBI-LCB1 |
| **NL_13** | 0 | *S. Epidermidis* | Yes | Yes | ALL/CC/UTI | MBI-LCB2 |
| **NL_14** | 7 | *P. Aeruginosa* | No | No | ALL/UTI | BSI other |
| **NL_15** | 6 | *S. Epidermidis - E.faecium* | No | No | ALL/CC/UTI - ALL/MBI/UTI | MBI-LCB2 + MBI-LCB1 |
| **NL_16** | 5 | *S. Warneri* | No | No | ALL/CC/UTI | MBI-LCB2 |
| **NL_17** | 4 | *S. Aureus/S. Epidermidis* | No | Yes (S. aureus) | ALL/UTI - ALL/CC/UTI | MBI-LCB2 + BSI other |
| **NL_18** | 14 | *Stenotrophomonas maltophila* | No | Yes | ALL/UTI | BSI other |
| **NL_19** | 5 | *S. Epidermidis* | No | Yes | ALL/CC/UTI | MBI-LCB2 |
| **NL_20** | 6 | *K. Pneumoniae* | No | Yes | ALL/MBI/UTI | MBI-LCB1 |
| **NL_21** | 0 | *MSSA E coli ESBL* | Yes (MSSA, E. coli) | Yes (MSSA, E. coli) | ALL/UTI - ALL/MBI/UTI | MBI-LCBI1 + BSI other |
| **NL_22** | 0 | *P. Aeruginosa/S. Salivarus* | No | No | ALL/UTI - ALL/CC/MBI/UTI | MBI-LCBI1 + BSI other |
